# Manual of Intragastric Technique

**Published:** 1903-12

**Authors:** 


**Manual of Intragastric Technique.** By George Herschell, M.D. Pp. vi., 166. London: Henry J. Glaisher. 1903.

To a very large number of medical practitioners the recently introduced manipulations for the diagnosis and treatment of diseases of the stomach are still unknown. This is, perhaps, due to the fact that the shorter contributions by gastro-therapeutic experts lack the detailed descriptions that are essential for clearly apprehending not only what to do, but how to carry out the methods in actual practice, while on the other hand the larger treatises are too full of detail for the novice. Dr. Herschell has had the advantage of a large practical experience, and has a rare faculty of conveying to the reader most clearly and concisely the results of such experience, so that he may carry out all the manipulations himself. He seems to anticipate all the difficulties that may arise, and tells us how to avoid them. Chapter I. consists in a description of the best form of stomach tube, and how to pass it and when not to pass it. The "diagnostic inflation of the stomach," the "extraction of the stomach contents," and the "examination of the stomach contents," each receives a chapter. "Lavage of the stomach," "some intragastric methods of treatment," and the "application of electricity to the stomach" occupy remaining chapters.

The book is printed in large clear type, the text being rendered clearer and more interesting by several illustrations of the most approved forms of apparatus. We can strongly recommend this small and eminently practical manual.

